# The Effect of “Synkavit” Administration Combined with Irradiation upon Rabbits and Rats

**DOI:** 10.1038/bjc.1954.54

**Published:** 1954-09

**Authors:** B. Jolles, J. O. Laws


### THE EFFECT OF "SYNKAVIT" ADMINISTRATION COMBINED WITH IRRADIATION UPON RABBITS AND RATS.

B. JOLLES AND J. O. LAWS

*From the Department of Radiotherapy, General Hospital, Northampton, and the Department of Experimental Pathology and Cancer Research, University of Leeds.*

Received for publication July 23, 1954.

AMONGST the attempts to improve the results in radiotherapy of some types of cancer, especially those in which results are unsatisfactory, by the ancillary use of chemical agents designed to act as radiosensitizers, the work of Mitchell (1948) has been most consistently and thoroughly pursued. After many clinical trials Mitchell (1950, 1952, 1953) has shown that the administration of tetrasodium 2-methyl-1 : 4-napthohydroquinone diphosphate (synthetic vitamin K derivative, Synkavit, Roche) to patients with advanced malignant disease improves significantly the survival results of treatment. Laboratory tests with tissue cultures and animal experiments, with a large series of chemical compounds (Mitchell and Simon-Reuss, 1952*a*, 1952*b*) have shown the superiority of this temporarily solubilised quinol.

Clinical trials with the compound were made in this department, and intramuscular and later intravenous injections of 100 mg. of Synkavit were given daily to patients as a coadjuvant to radiation treatment for advanced carcinomas. Some 3½ years ago a striking improvement in the general condition of the majority of 100 patients undergoing radiotherapy for advanced cancer, to whom Synkavit injections were given had been observed. This might be attributed to an increased tolerance of radiation, and this possibility led to a series of animal experiments in which a comparison was made between the lethal effects of X-rays in animals receiving Synkavit prior to and after irradiation with control animals which received X-rays and a series of injections of saline, and animals which received X-rays and no injections at all.

The work here described is concerned with the animal experiments only as the clinical material available does not allow data regarding the effectiveness of Synkavit as a coadjuvant of radiotherapy as yet to be presented.

### MATERIALS AND METHODS.

Sixty-five rabbits and 75 rats were used in these experiments.

The rabbits were Old English, farm bred, and of a non-homogeneous strain. Attempts were always made to use in sets of experiments to be compared animals of similar age and body weight.

The rats were of the "Birmingham Strain," obtained by the courtesy of Dr. A. M. Mandl, Department of Anatomy, Birmingham University. Their weights at commencement of experiment were 200 $\pm$ 10 g. and were taken daily or every other day. Food and water were supplied *ad libitum* otherwise the general maintenance and handling of the animals was standardised.

Thirty-five rabbits and 37 rats were given subcutaneous injections of Synkavit, the amounts given to rabbits ranging from 10–50 mg. daily, but constantly 10 mg. in the case of rats. The remainder served as controls. The drug was given in some cases for a few days before the delivery of the X-ray dose and continued for a time varying from 10–17 days. The dose of X-rays given in the case of rabbits ranged from 600–1500 r. There were three groups of 26, 24 and 25 rats which received respectively 480 r, 580 r and 650 r.

All the rabbits and all the rats were exposed to a beam of radiation covering the whole body. The physical factors were 180 kV 10mA. 50 cm. F.S.D. with a H.V.L. varying according to the filtration, which was either the inherent tube shield filtration alone or 0·5 mm. Cu. (H.V.L. 0·85 mm. Cu.). The irradiation was carried out with the tube head and the applicator inverted, with the box containing the animals standing on top of the applicator end, the incident beam falling on the ventral surface of the animals. The rabbits were not anaesthetised but kept still by padding the box and its partitions. Autopsy was performed in almost all animals dying as a result of irradiation, and those which were killed after they had survived a period of 30–50 days. Sections were taken of the lung, spleen, adrenals, liver and testes in almost all cases.

### RESULTS.

*Rabbits.*

The difference in survival rates between the Synkavit plus X-rays' series of rabbits with the series of rabbits which received X-rays alone was not significant when the assessment was made on the whole series of 35 rabbits which received Synkavit injections. Fourteen out of 35 rabbits survived the 30 days' period, the remaining 21 survived on an average 8 days after a dose of 800–1500 r had been given in conjunction with Synkavit. The amount of the latter varied according to the number of injections given, which in many animals was cut short by death.

In a control series 5 out of 12 survived similar doses of X-rays without Synkavit, the remaining 7 dying within an average of 7·5 days. The weight curve of these animals differed in the two series considerably. In the Synkavit group (Fig. 1) the majority maintained their weight fairly well throughout the observation period or gained ground slightly. This was particularly so in the case of the rabbits which received Synkavit injections for a few days prior to exposure to X-rays. In the series of rabbits exposed to X-rays without Synkavit (Fig. 2) there was a consistent drop in the weight curve, with the exception of one surviving rabbit which gained some 10 per cent in weight over a period of 30 days. The Synkavit rabbits were on the whole more lively and taking food more liberally than the animals without Synkavit.

*Rats.*

The 75 rats used in these experiments were dealt with in three separate series.

*Group I.*—Thirteen rats received 10 mg. of Synkavit injections daily (7 rats for 4 days and 6 rats for 5 days) prior to exposure to a dose of 580 r and subsequently daily until their death, and a group of 13 rats received a similar dose of X-rays to the whole body without any injections. In the Synkavit group there was 1 survivor and in the non-Synkavit series there were no survivors at 30 days.

While in the Synkavit series 3 deaths occurred within 15 days there were no deaths before 21 days in the X-rayed group not receiving the compound (Fig. 3).

*Group II.*—Twenty-four rats were exposed to a dose of 650 r to the whole body. Of these, 11 were given injections of 10 mg. of Synkavit daily, and 13 were given daily injections of 1 ml. of sterile normal solution of saline. There were no 30–day survivals in the whole series (Fig. 4). Of the 11 Synkavit rats, 7 died at 4 days, 2 at 6 days, 1 at 7 days and 1 at 9 days after irradition. In the saline group the deaths occurred : 1 rat at 3 days, 5 at 4 days, 2 at 6 days, 2 at 7 days, 1 at 8 days, 1 at 9 days and 1 at 15 days.

FIG. 1.—Body weight and survival (S) curves of irradiated rabbits which received Synkavit injections.

*Group III* (Fig. 5).—A group of 25 rats were exposed to a dose of 480 r to the whole body. Of these 9 received seven and 4 received five injections, 10 mg. daily, of Synkavit prior to exposure to X-rays and eight to eleven daily injections after exposure. Eight rats received six pre-X-ray treatment and 10 post-treatment injections of 1 c.c. of sterile normal solution of saline and 4 received no injections whatsoever. In the group of rats which received saline injections there were no deaths at 31 days after irradition. In the group of rats which received Synkavit injections there was only 1 survival at 31 days, the remainder dying within 12–16 days. The 4 control (X-ray only) rats survived 31 days. As regards the weight curve in the 580 r series, all the Synkavit rats showed a loss which varied from 15 to 30 per cent, of their body weight, while in the non-Synkavit series there was also a consistent loss of weight though perhaps to a lesser extent. In the 650 r series the death of the animals occurred so rapidly after the irradiation

FIG. 2.—Body weight and survival curves of irradiated rabbits which received no injections.

FIG. 3.—Survival times of 26 rats of which 13 received Synkavit injections prior to and after irradiation (580 r) and 13 were irradiated but did not receive any injections.

that few weight data are available. The same applies to the 480 r Synkavit rats while in the saline and control 480 r rats a consistent drop in weight was noted.

*Microscopical.*

The spleen and adrenals of both rats and rabbits were examined in these experiments. The adrenal has been comparatively little studied, and yet is considered

FIG. 4.—Survival times of 24 rats (12 with Synkavit and 12 with saline injections) given a dose of 650 r.

FIG. 5.—Survival times of 25 rats (13 with Synkavit, 8 with normal saline injections and 4 without any injections) exposed to 480 r.

in the recent literature to play some part in the syndrome of irradiation sickness (Porter, 1952) and in the repair of tissue damage following irradiation (Craver, 1948). The importance of adrenal hormones in maintaining many vital functions also suggest a potential connection between this organ and the widespread changes present after irradiation.

*Spleen.*

The changes in the spleen followed the same pattern in both species and were of the type previously described in the mouse by Jacobson *et al.* (1950). The lymphoid follicles disappeared rapidly in the first 3 weeks. Those surviving for longer periods however often presented a relatively normal appearance in the lymphoid nodules, due to regeneration following, perhaps, less initial damage, though this can only be surmised. In many of the animals dying in the earlier period there were further changes of an atrophic nature, in some cases little except blood vessels and fibrous tissues being seen. Iron containing pigment was often prominent in these specimens. There was in general no difference between animals given Synkavit and the controls dying after the same period.

*Adrenal.*

The histopathology of the adrenal following direct irradiation has received surprisingly little attention, especially from modern workers. As Rhoades remarked in 1948 the material available is slight and mostly contradictory. The chief papers are those of Frey (1928), Engelstad and Torgersen (1937) and Warren (1943). The first concerns the guinea-pig and under the conditions of the experiment the author concluded that there was no direct effect of the radiation on the cortex. Engelstad and Torgersen (1937), working with rabbits, decided on the other hand that with high dosage there was a marked effect leading to degeneration of the cortex over a period starting a few days after the irradiation, and, in surviving rabbits, progressing even for 2 or 3 months. They concluded that the radiosensitivity of the cortical tissue approximates to that of the skin. The medulla showed little change. These observations were confirmed by Warren (1943) who emphasized that it is difficult to distinguish minimal changes from those of secondary radiation effects. There appears to be no previous study of the rat adrenal.

*Rabbit.*—In general the findings in these species are similar to those of Engelstad and Torgersen (1937). The changes in the cytoplasm consist of loss of lipoid at an early stage and in some cases a marked basophilia. This latter tends to disappear but lipoid is slow to reappear in comparison with what is usually seen in secondary adrenal involvement (Nizet, Heusghem and Herve, 1949) often being small in amount at the end of 31 days. The nuclear changes affect particularly the zona fasciculata extending up to the medulla (the zona reticularis is hardly distinguishable in the rabbit). The nuclei may undergo any of the commonly seen forms of degeneration, pyknosis, fragmentation or loss of basophilia followed by dissolution. Apart from occasional initial hyperaemia there is no inflammatory reaction and one has the impression that some of the degenerated cells remain *in situ* almost indefinitely. How much function may remain in spite of the nuclear changes is an interesting point for further study.

In contrast to the constant degenerative changes in the inner zones of the cortex, the zona glomerulosa in the rabbit shows comparatively slight damage.

Both cytoplasm and nuclei appear relatively normal. No mitosis was seen in the rabbit material but in the presence of some of the relatively normal cells, its occurrence cannot be excluded on the present evidence.

*Rat.*—The normal rat adrenal differs from that of the rabbit in that the three zones are well demarcated, not only by the general cellular arrangement, but also by the nuclear character. In the zona glomerulosa the nuclei have the chromatin well dispersed with a slight concentration only on the nuclear membrane. In the fasciculata there is a tendency for it to be accumulated in small granules and this becomes very marked in the reticularis where it appears to be present mainly in masses on the nuclear membrane, giving a " clock face " appearance in many.

In the irradiated animals it was found that, unlike what had been seen in the rabbit, all zones seemed equally affected, showing cytoplasmic and nuclear changes. The cytoplasmic damage was similar to that seen in the rabbit. The nuclear alterations varied according to the normal patterns seen in the various zones. In the reticularis pyknosis was the rule ; in the fasciculata, either pyknosis or a loss of basophilia and " fading "; in the reticularis a breaking up of the nucleus, the stroma disappearing and leaving the chromatin particles scattered.

In animals dying in the first few days after irradiation, hyperaemia in the zona reticularis was common. As in the rabbit no evidence of an inflammatory reaction was seen at any time. Mitosis was in general absent but a few mitotic figures were seen in 2 rats dying on the 29th and 31st days. They were all in the outer part of the zona fasciculata.

In neither species was there any unequivocal evidence of the effect of Synkavit. In the rat, however, the general impression was one of greater damage in those receiving the compound than in the controls.

### DISCUSSION.

(1) In rabbits the administration of Synkavit prior to and after irradiation has shown slight increase in tolerance of irradiation, and although the difference in the mortality rate of rabbits and rats is not significant, the weight curve and the well-being of the animals suggested an increased tolerance of radiation in this species. In rats an opposite effect was found (Mitchell, 1951 ; Jolles, 1952). As, however, the amount of Synkavit given to rats was approximately twice as big per body weight as that given to rabbits it is quite reasonable to expect more obvious effects in the former. The findings in rabbits provide useful evidence that the amounts given to patients are not excessive, and are not likely to influence the radiosensitivity of the normal tissues which has to be avoided in clinical trials aiming at radiosensitization of tumours. The histological changes in the adrenals stained solely with haematoxylin and eosin can clearly give no more than a hint of the changes which take place in the irradiated animals. Nevertheless, enough has been found to suggest that a more detailed study of adrenal pathology and function might be fruitful in elucidating the mechanism of radiation effects. The difference in the sites of damage in the adrenals of rats and rabbits, with the comparative sparing of the zona glomerulosa in the latter, invites speculation as to whether the greater resistance of this animal to whole body radiation might be associated with a continued secretion of salt retaining hormones from this zone. Species differences in adrenocortical secretion which are genetically determined and which cannot at present be related to any known differences of adrenocortical function have been investigated by Bush (1953). He deals however only with 3 keto-steroids in which there is no difference between rat and rabbit, although these two differ considerably from other species studied. The problem is clearly a complex one but the existence of these species' differences may in fact offer useful clues to the changes which follow irradiation. In this connection a clear distinction must be made between the direct damage to the adrenal in whole body irradiation and indirect adrenal stimulation. The salt retention found by Cori, Pucker and Goltz (1923) in sickness following localised therapeutic radiation in humans, and the transient loss of lipoid from the adrenal cortex of rabbits demonstrated by Nizet, Heusgham and Herve (1949) are almost certainly, as suggested by the latter authors, an indirect effect. The long persistence of many cells with abnormal nuclei also raises the question as to the relation of morphology and function under these circumstances.

(2) The histological findings of the effects of Synkavit are not disquieting in view of the fact that the main demonstrable effect of this compound is mitotic inhibition (Mitchell, 1949). It is perhaps not surprising that the histological appearance of tissue already showing nuclear damage and mitotic arrest from the effect of the X-rays should be little altered by the chemical. Nevertheless the suggestion of greater damage in the adrenals of rats receiving Synkavit makes it possible that this may at least have contributed to the deleterious effect of this compound in this species.

Although it is hazardous to generalise from the described experiments, the fact that patients suffering from malignant tumours who receive daily injections of Synkavit while undergoing radiotherapy stand treatment better induces one to venture an opinion that the administration of this compound in humans produces a response similar to that found in rabbits rather than that of rats. In this context, however, it has to be borne in mind that a selective concentration of the drug in tumours has been shown by Mitchell (1950) by means of ultra-violet microphotography and that the exhibition of Synkavit in normal individuals probably produces effects different from those to be expected in tumour bearing patients receiving treatment to a part of the body only.

The relatively rare occurrence of abdominal or thoracic haemorrhage as seen post mortem of Synkavit treated animals was noteworthy. The results in rats which received daily injections of 1 ml. of 0·9 per cent NaCl. solution subsequent to the exposure to X-rays are in agreement with similar findings of Ellinger and Blagg (1952) in mice.

### SUMMARY.

Experiments are described in which a comparison was made between the lethal effects of X-rays in rabbits and rats receiving Synkavit prior to and after irradiation, with control animals which received X-rays and a series of injections of saline and animals which received X-rays and no injections at all.

In rabbits the administration of Synkavit prior to and after irradiation has shown a slight increase in tolerance of irradiation, while in rats an opposite effect was found. The histological changes in the spleen and adrenals of the animals in the two series are described and the question of species differences are discussed. The findings in rabbits provide useful evidence that the amount given to patients is not excessive and is not likely to influence the radiosensitivity of the normal tissues, which has to be avoided in clinical trials aiming at radiosensitization of tumours.

The authors are indebted to the British Empire Cancer Campaign for a grant for technical assistance.

The work of Mr. G. B. Dun, research technician at the Department of Radiotherapy, General Hospital, Northampton, who has materially contributed to the work described is gratefully acknowledged.

Thanks are also due to Dr. F. Wrigley, Dr. A. L. Morrison, and Dr. J. F. T. Allison of Roche Products Limited, Welwyn Garden City, for supplies of Synkavit.

### REFERENCES.

Bush, I. E.—(1953) *J. Endocrinol.*, **9**, 95.
Cori, K. F., Pucker, G. W., and Goltz, H.—(1923) *Amer. J. Roentgenol.*, **10**, 738.
Craver, B. N.—(1948) *Ibid.*, **59**, 404.
Ellinger, F., Blagg, J. W.—(1952) Research Report. Naval Medical Research Inst., Naval Medical Center, Bethesda, Project NM. 006.012.05.09.
Engelstadt, R. B., and Torgersen, O.—(1937) *Acta radiol., Stockh.*, **18**, 671.
Frey, H.—(1928) *Ibid.*, **9**, 23.
Jacobson, L. O., Simmons, E. L., Marks, E. K., Robson, M. J., Bethard, F. W., and Gaston, E. O.—(1950) *J. Lab. clin. Med.*, **35**, 746.
Jolles, B.—(1952) *Rep. Brit. Emp. Cancer Campgn.*, **30**, 325.
Mitchell, J. S.—(1948) *Brit. J. Cancer*, **2**, 351.—(1949) *Rep. Brit. Emp. Cancer Campgn*, **27**, 214.—(1950) *Ibid.*, **28**, 217.—(1951) *Ibid.*, **29**, 192.—(1952) *Ibid.*, **30**, 239.—(1953) *Brit. J. Cancer*, **7**, 313.
*Idem* and Simon-Reuss, I.—(1952*a*) *Ibid.*, **6**, 305.—(1952*b*) *Ibid.*, **6**, 317.
Nizet, E., Heusghem, C., and Herve, A.—(1949) *C.R. Soc. Biol., Paris*, **143**, 876.
Porter, E. C.—(1952) *Radiology*, **58**, 246.
Rhoades, R. P.—(1948) 'Histopathology of Radiation.' (Ed. W. Bloom). McGraw Hill, National Nuclear Energy Series.
Warren, S.—(1943) *Arch. Path.*, **35**, 304.


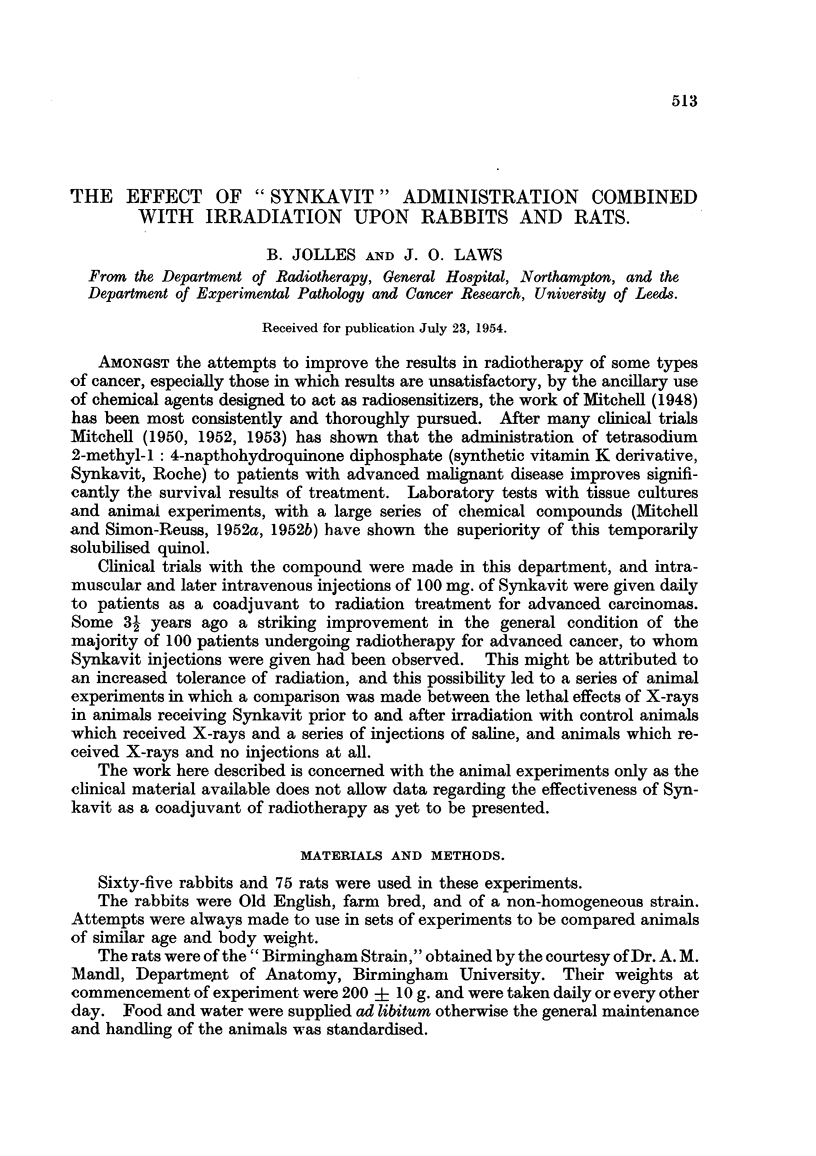

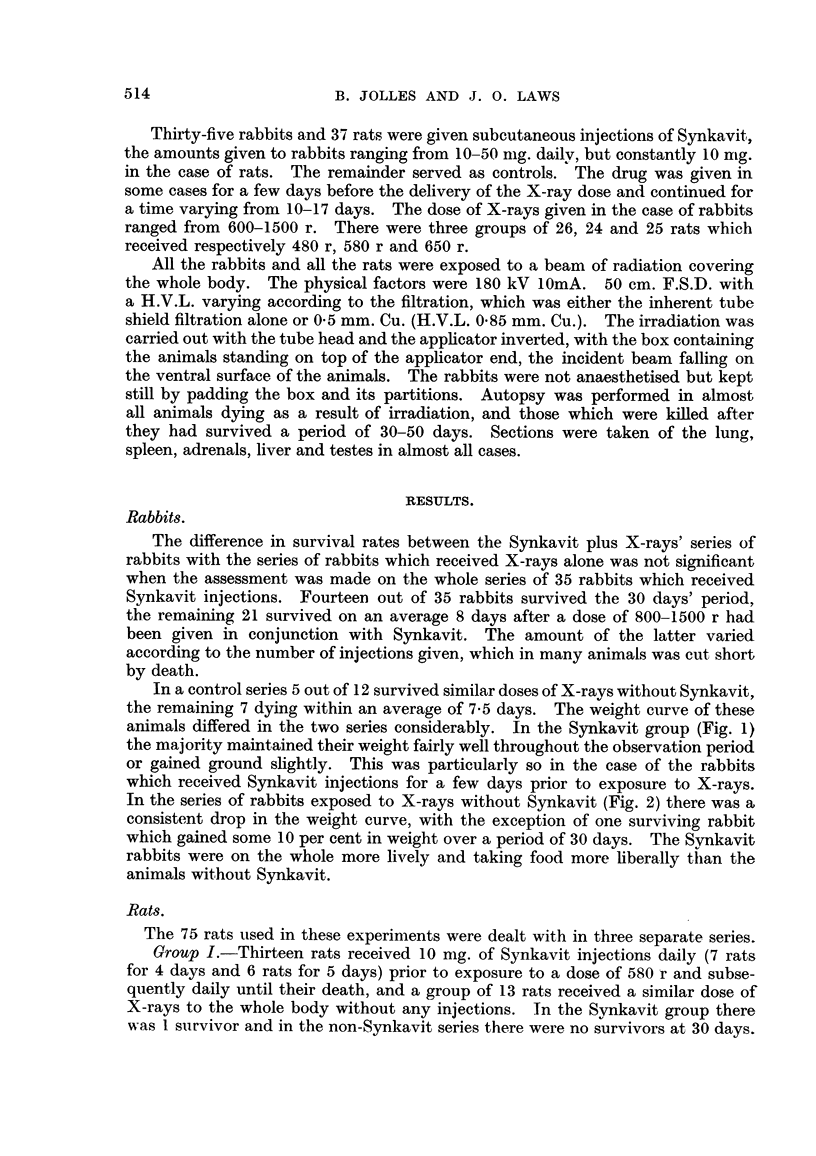

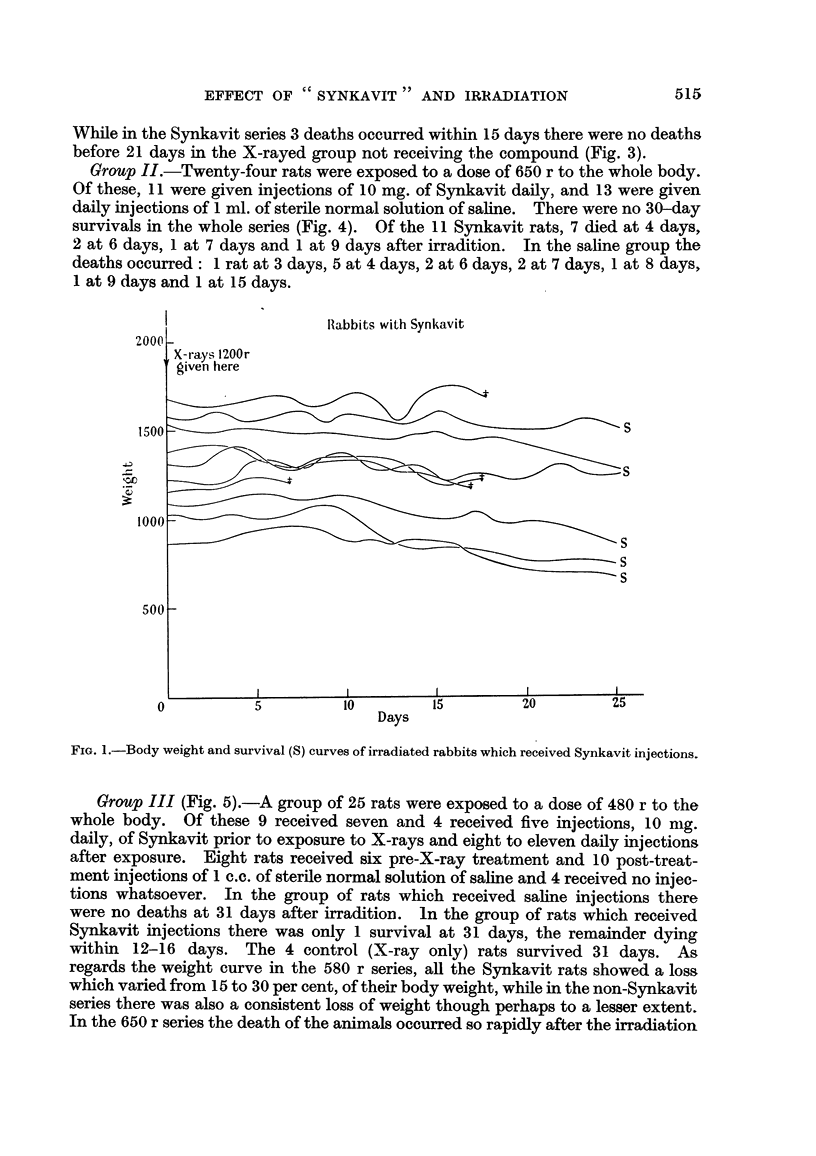

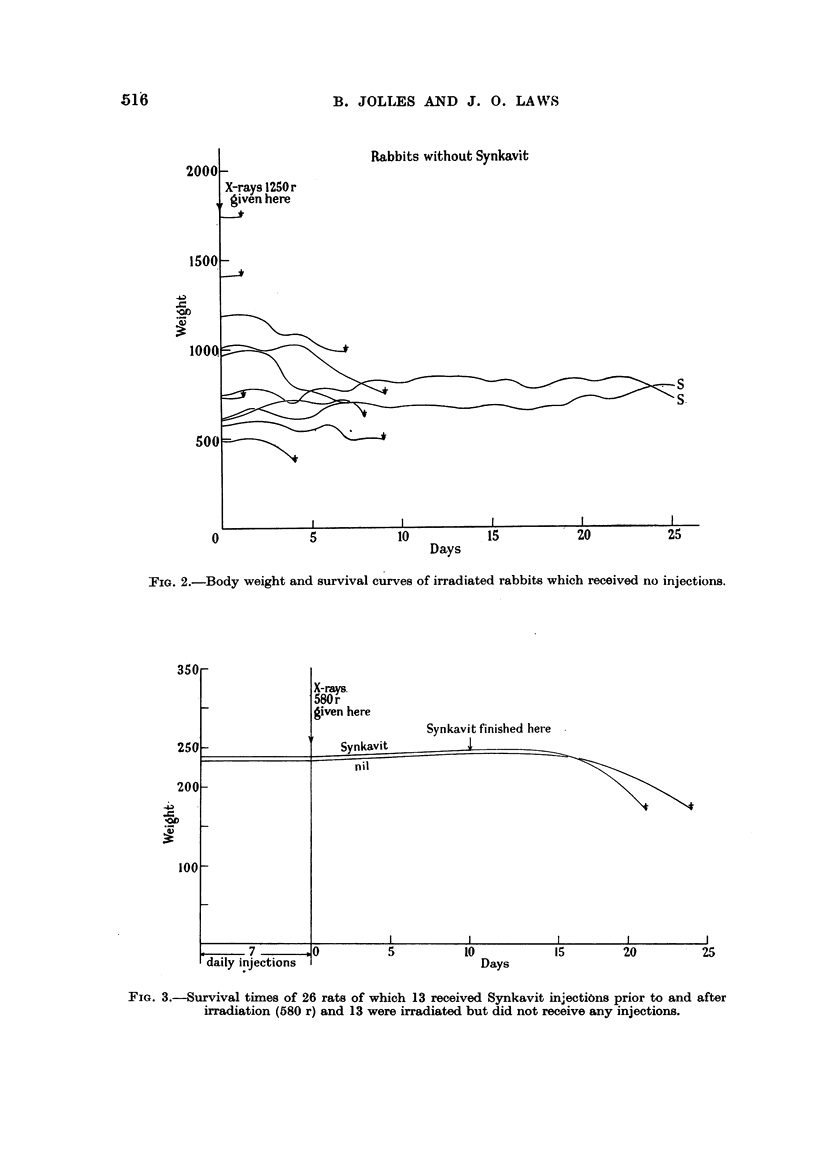

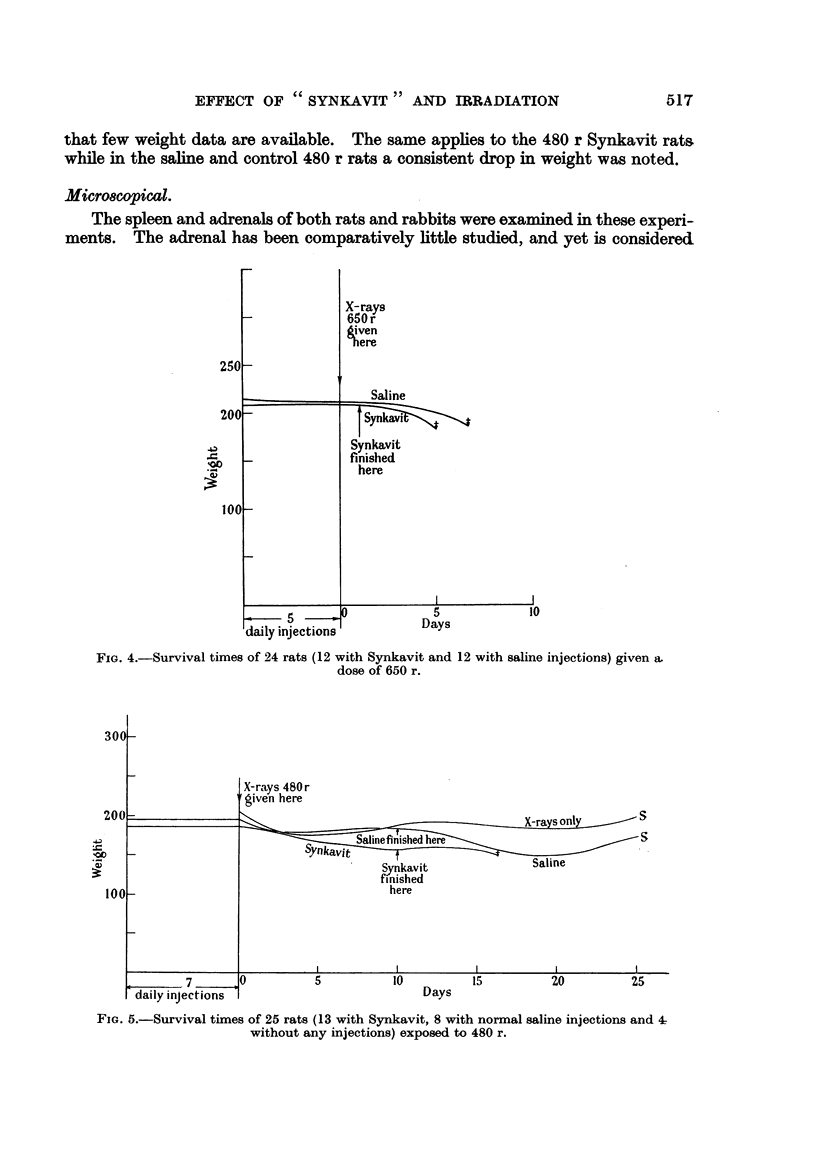

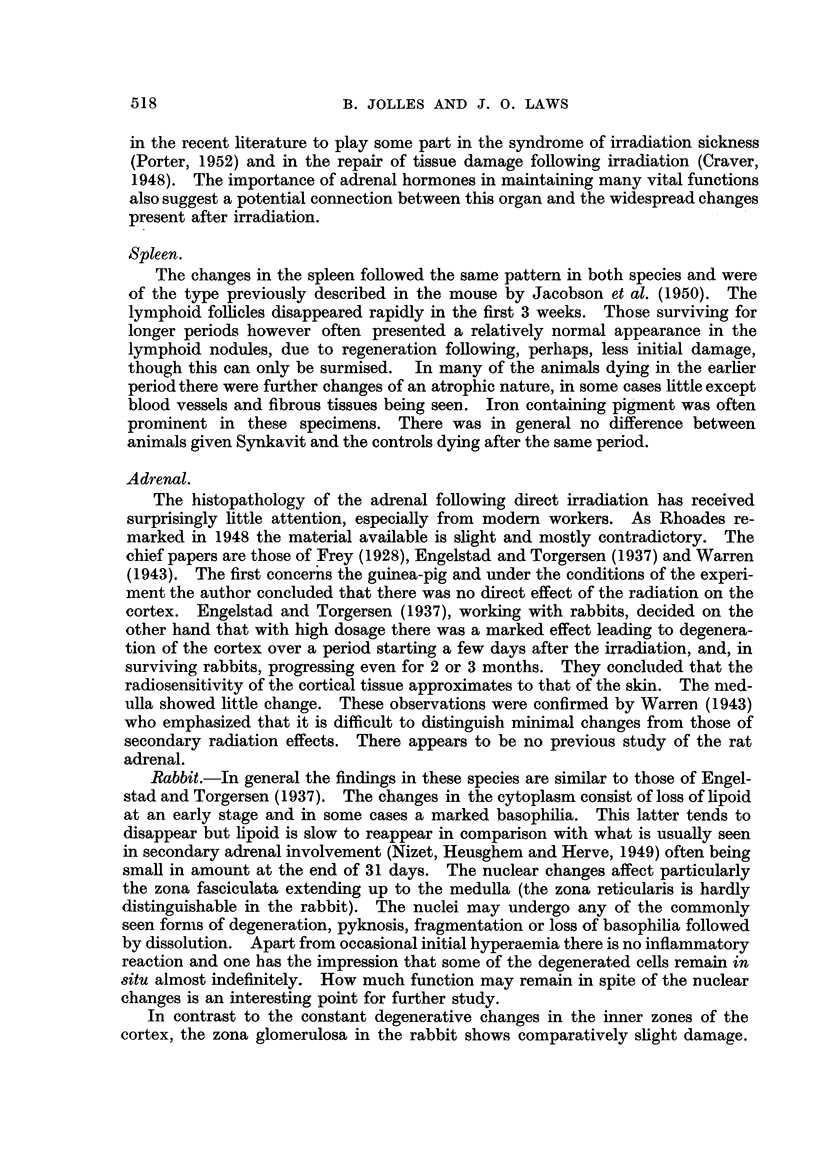

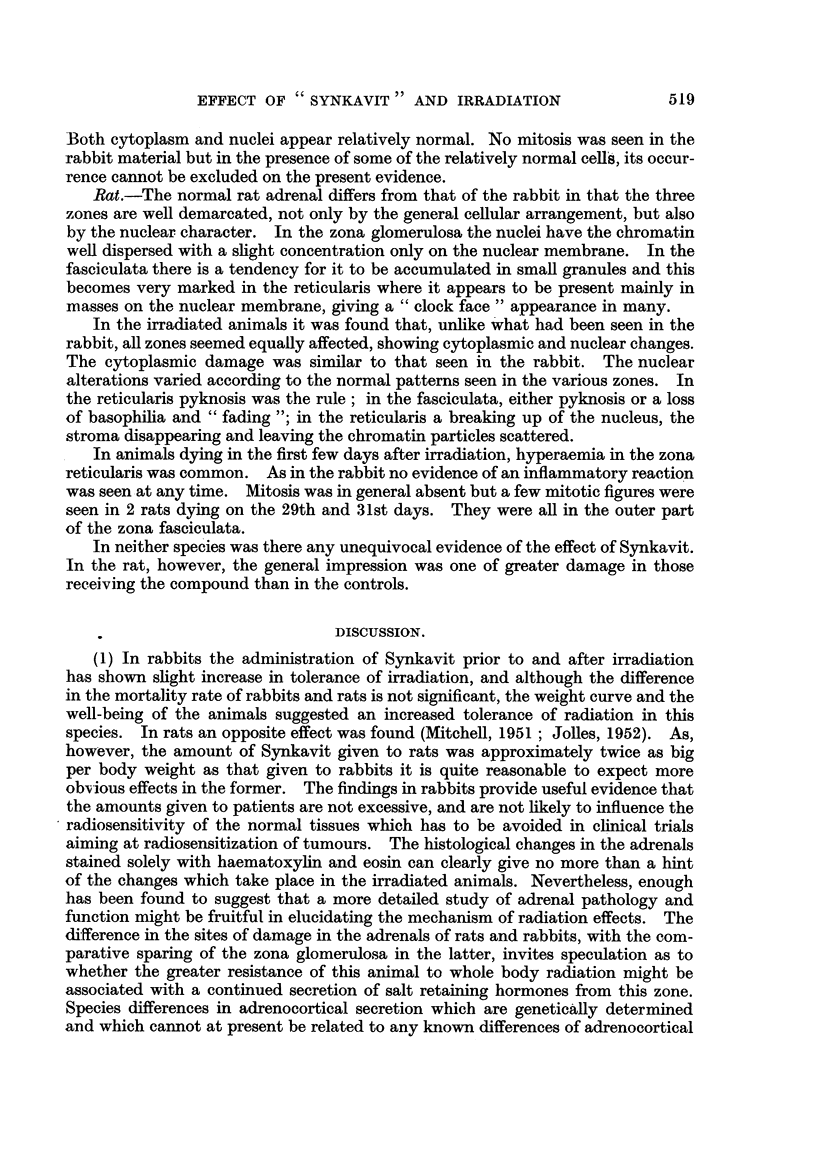

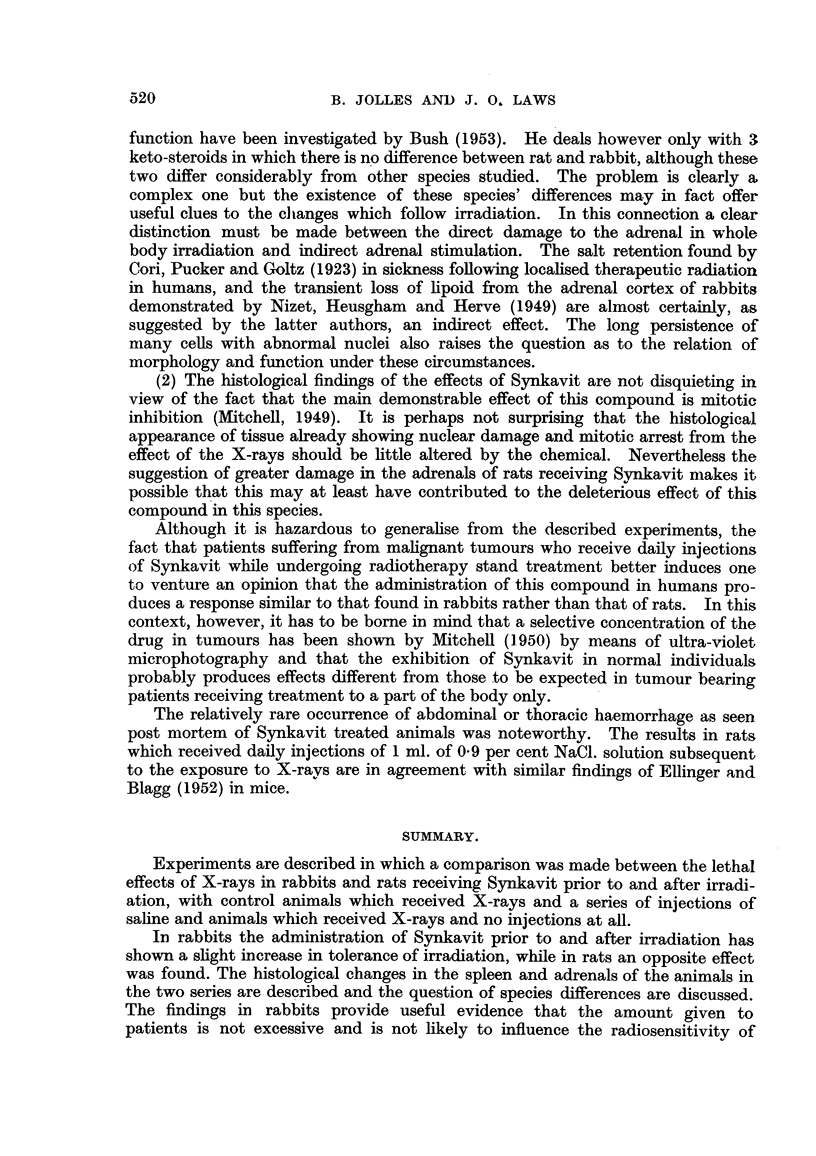

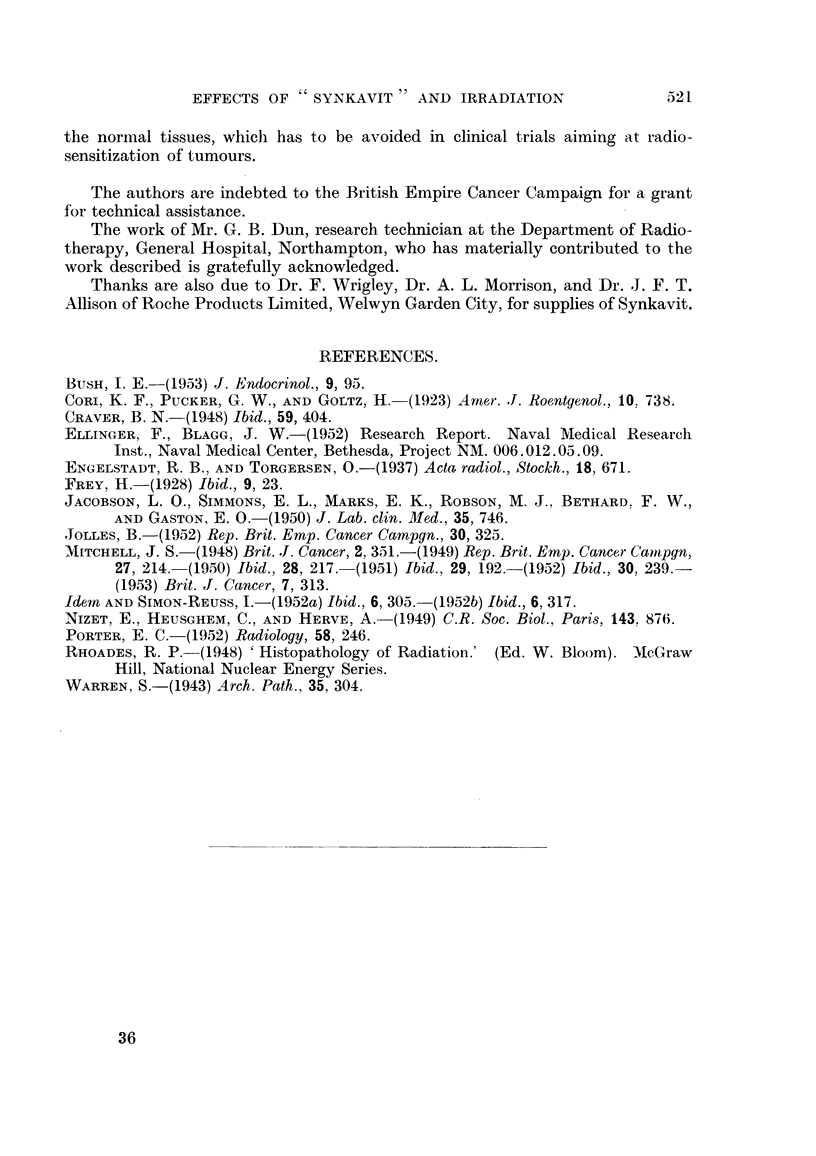


## References

[OCR_00515] JACOBSON L. O., SIMMONS E. L., MARKS E. K., ROBSON M. J., BETHARD W. F., GASTON E. O. (1950). The role of the spleen in radiation injury and recovery.. J Lab Clin Med.

[OCR_00528] PORTER E. C. (1952). Relationship between the adrenal cortex and radiation sickness; a review of the literature and a presentation of new data.. Radiology.

